# Nitrous oxide emission related to ammonia-oxidizing bacteria and mitigation options from N fertilization in a tropical soil

**DOI:** 10.1038/srep30349

**Published:** 2016-07-27

**Authors:** Johnny R. Soares, Noriko A. Cassman, Anna M. Kielak, Agata Pijl, Janaína B. Carmo, Kesia S. Lourenço, Hendrikus J. Laanbroek, Heitor Cantarella, Eiko E. Kuramae

**Affiliations:** 1 Department of Microbial Ecology, Netherlands Institute of Ecology, 6708 PB, Wageningen, Netherlands; 2 Soils and Environmental Resources Center, Agronomic Institute of Campinas, P.O. Box 28, 13012-970, Campinas, SP, Brazil; 3 Environmental Science Department, Federal University of São Carlos, 1852-780, Sorocaba, SP, Brazil; 4 Institute of Environmental Biology, Utrecht University, Netherlands

## Abstract

Nitrous oxide (N_2_O) from nitrogen fertilizers applied to sugarcane has high environmental impact on ethanol production. This study aimed to determine the main microbial processes responsible for the N_2_O emissions from soil fertilized with different N sources, to identify options to mitigate N_2_O emissions, and to determine the impacts of the N sources on the soil microbiome. In a field experiment, nitrogen was applied as calcium nitrate, urea, urea with dicyandiamide or 3,4 dimethylpyrazone phosphate nitrification inhibitors (NIs), and urea coated with polymer and sulfur (PSCU). Urea caused the highest N_2_O emissions (1.7% of N applied) and PSCU did not reduce cumulative N_2_O emissions compared to urea. NIs reduced N_2_O emissions (95%) compared to urea and had emissions comparable to those of the control (no N). Similarly, calcium nitrate resulted in very low N_2_O emissions. Interestingly, N_2_O emissions were significantly correlated only with bacterial *amoA*, but not with denitrification gene (*nirK*, *nirS*, *nosZ*) abundances, suggesting that ammonia-oxidizing bacteria, via the nitrification pathway, were the main contributors to N_2_O emissions. Moreover, the treatments had little effect on microbial composition or diversity. We suggest nitrate-based fertilizers or the addition of NIs in NH_4_^+^-N based fertilizers as viable options for reducing N_2_O emissions in tropical soils and lessening the environmental impact of biofuel produced from sugarcane.

Agriculture is the main anthropogenic source of N_2_O emissions, which are predicted to increase as nitrogen fertilizer use increases worldwide to meet the global food demand^1^. Currently, N_2_O emissions derived from N fertilizers account for up to 40% of total greenhouse gases (GHG) emissions in ethanol production from sugarcane^2^. High N_2_O emissions can negate the benefits of GHG reduction of biofuels used to replace fossil fuels^3^.

Emissions of N_2_O from soils occur mainly through nitrification and denitrification processes. These processes are carried out by autotrophic and heterotrophic microorganisms belonging to Bacteria, Archaea and Fungi divisions^4^,^5^,^6^. Other N transformations such as nitrifier denitrification, dissimilatory reduction of NO_3_^−^ to NH_4_^+^, chemo-denitrification and co-denitrification may also produce N_2_O. Despite considerable knowledge of the processes evolving N_2_O, the prevalence of these processes in tropical soils has only begun to be addressed.

The denitrification process has been demonstrated to contribute more to N_2_O emissions than nitrification at soil moisture levels above 75% of the water-filled pore space (WFPS); however, nitrification has been observed to be more prevalent in soil at 60% WFPS^7^. High correlation between N_2_O emissions and bacterial *amoA* and *nirK* abundances are observed^8^, suggesting that both nitrification and denitrification and/or nitrifier denitrification processes are responsible for N_2_O emissions when cattle urine is applied to soils with 100 and 130% of water-holding capacity.

In the central-west and southeast regions of Brazil, about 80% of the land area is cultivated with sugarcane^9^. The dominant soils in these regions are Red Latosols (Hapludox), which are highly weathered, deep and well-drained soils^10^. Here, we expected that denitrification would be low because the optimal conditions are at least 60% WFPS. Though high levels of rainfall and anaerobic conditions in soil micropores may increase the contribution of denitrification to N_2_O emissions^11^, we predicted based on the high soil drainage that nitrification would be the major pathway contributing to N_2_O emissions. In this case NH_4_^+^-based fertilizer would result in higher N_2_O emissions than those from NO_3_^−^-based fertilizers in these soils. Up to date, this process has not been shown for these types of soils grown with sugarcane.

The Intergovernmental Panel on Climate Change (IPCC) estimates that 1% of N applied is emitted as N_2_O as default value^12^. However, in practice, different amounts of N_2_O are emitted depending on N fertilizers and soil types, and environmental conditions^13^,^14^,^15^. Therefore, experiment-based nitrogen management is an important tool to decrease N_2_O emissions and to reduce the environmental impact of agricultural practices^15^.

Urea is the most widely used fertilizer in the world, and generally has been linked to higher N_2_O emissions compared with other N sources^14^. One way to reduce N_2_O emissions is the addition of specific nitrification inhibitors (NIs) such as dicyandiamide (DCD), 3,4 dimethylpyrazone phosphate (DMPP), nitrapyrin, and others with urea fertilization^15^,^16^. These nitrification inhibitors block the enzyme ammonia monooxygenase in the first step of nitrification^17^. The gene encoding this enzyme is *amoA*, present in ammonia-oxidizing bacteria (AOB) and archaea (AOA). Several studies indicate that DCD and DMPP reduce AOB or AOA gene abundances, depending on which microorganism was prevalent^8^,^18^,^19^,^20^. DCD has been reported to reduce also the abundance of *nirK*, probably because AOB abundances are correlated with *nirK* abundances, implying reduced nitrifier denitrification abundances^8^.

To our knowledge, there are no studies identifying the main microbial processes, the effect of different fertilizers on N_2_O emissions, and the impact of different N fertilizers on the microbial community in tropical soils grown with sugarcane. Therefore, the goals of this study were to (i) determine the main microbial process responsible for the N_2_O emissions, (ii) evaluate the efficacy of enhanced-efficiency fertilizers, including nitrification inhibitors, in reducing N_2_O emissions, and (iii) determine the short-term effects of the fertilizer treatments on bacterial community composition and diversity.

## Results

### Greenhouse gases emissions

**Figure 1:**
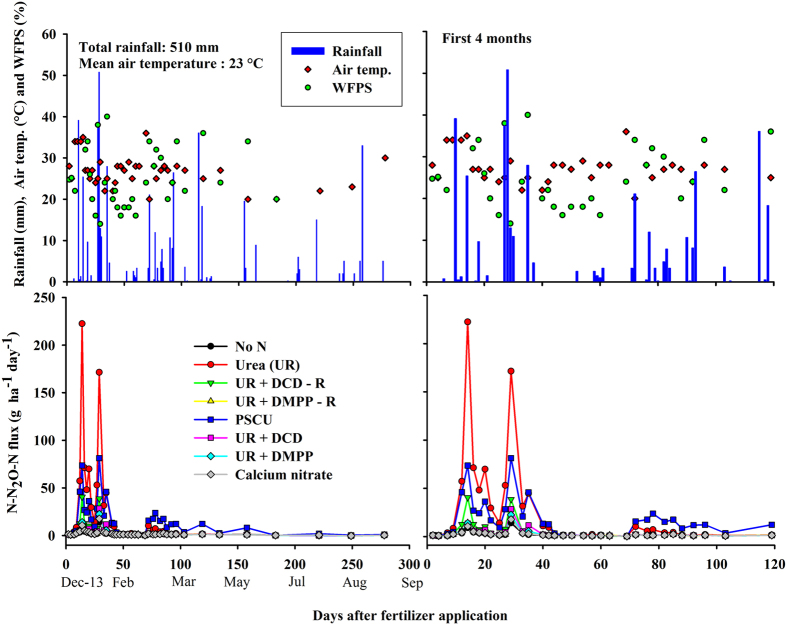
Rainfall, air temperature, water-filled pore space (WFPS) and nitrous oxide fluxes from Control (No N), urea (UR) with or without nitrification inhibitors (DCD and DMPP), polymer sulphur coated urea (PSCU) and calcium nitrate applied to sugarcane. R: reapplication of inhibitors in the same plots during the previous two cycles of the experiment. N fertilizers were applied on 13 December 2013.

**Figure 2:**
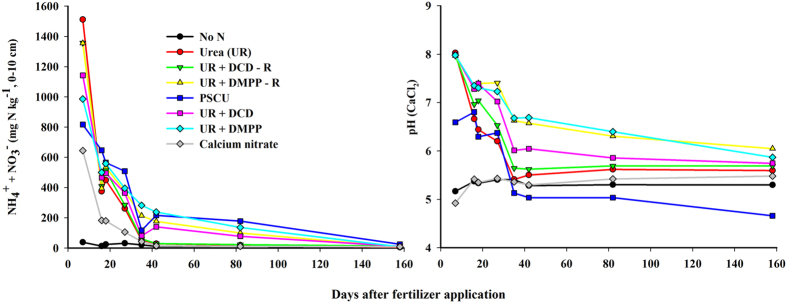
Soil concentration of NH_4_^+^-N + NO_3_^−^-N and pH from Control (No N), urea (UR) with or without nitrification inhibitors (DCD and DMPP), polymer sulphur coated urea (PSCU) and calcium nitrate applied to sugarcane. R: reapplication of inhibitors in the same plots during the previous two cycles of the experiment.

Emissions of N_2_O were low in the first 10 days after fertilizer application, with less than 10 g ha^−1^ day^−1^ of N emitted (Fig. 1). High N_2_O emission followed rain events coupled with high soil inorganic N availability (Fig. 2). The UR treatment had the highest peak of N_2_O emission, on the 14^th^ day, which corresponded to a loss rate of more than 200 g ha^−1^ day^−1^ of N. On the 29^th^ day, another high emission peak of about 170 g ha^−1^ day^−1^ occurred. Between these peaks, N_2_O emissions were still relatively high ranging from 15 to 70 g ha^−1^ day^−1^ in the UR treatment. The treatments UR+DCD, UR+DMPP, and calcium nitrate had smaller N_2_O fluxes than those of UR, showing emission levels similar to the control treatment (around 5 g ha^−1^ day^−1^ of N). Urea containing NIs (UR+DCD-R, UR+DMPP-R) had been reapplied in the same plots in the previous two years^16^. Emissions in the plots with repeated application of NIs were also low at ≤5 g ha^**−**1^ day^−1^ of N (Fig. 1). The controlled release fertilizer PSCU treatment showed lower N_2_O emission (80 g ha^−1^ day^−1^) compared to the UR treatment on the 14^th^ and 29^th^ days, but was similar to UR treatment levels afterwards, until the 50^th^ day. Between 70 and 120 days after fertilizer application, N_2_O emissions were greater in the PSCU treatment (between 10–20 g ha^−1^ day^−1^) compared to the other treatments (2 g ha^−1^ day^−1^) (Fig. 1).

Cumulated N_2_O emissions in the control treatment were equivalent to 0.3 kg ha^−1^ after 278 days. The UR treatment emitted more than 2.3 kg ha^−1^ of N_2_O-N, which corresponded to 1.7% of total N applied. The UR+DCD, UR+DMPP treatments resulted in considerable reductions in cumulated N_2_O emissions compared to UR, with emissions that did not differ from those of the control (Table 1). The reduction of N_2_O emissions by addition of NIs to UR varied from 88 to 97% (95% in average). The PSCU treatment resulted in cumulative emissions similar to those of UR. Calcium nitrate resulted in low N_2_O emissions that did not differ from those of the UR+DCD and UR+DMPP treatments or the control (Table 1).

**Figure 3:**
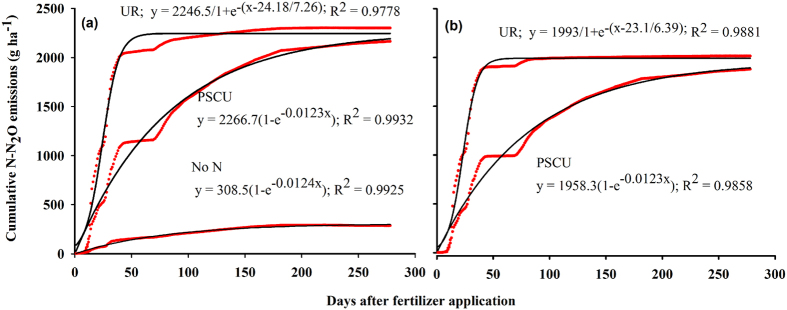
Cumulative N_2_O emission (red dots) and sigmoidal or exponential equations fitted (black lines) to data of urea (UR) and polymer sulphur coated urea (PSCU) applied to sugarcane. (**b**) Net UR and PSCU N_2_O emissions calculated by subtracting data of the control treatment.

Cumulative N_2_O emissions data were fit with sigmoidal or exponential equations for the control, UR and PSCU treatments. These models were not applied to the UR+DCD, UR+DMPP, and calcium nitrate treatments because in these treatments the N_2_O emissions did not differ from the control. The N_2_O emissions in the control treatment were low and whether included or excluded did not affect the model equations. The UR treatment achieved 90% of maximum N_2_O loss 40 days after fertilizer application. The N_2_O emission lasted longer with the PSCU treatment than with UR; 90% of total N_2_O emission was achieved 187 days after fertilization with PSCU (Fig. 3).

The total CO_2_ and CH_4_ emissions were around 6 t ha^−1^ and −600 g ha^−1^, respectively. For both CO_2_ emissions and CH_4_ consumption, no differences between the treatments were observed (Table 1).

### Soil analysis

In the first soil sampling seven days after fertilizer application, the calcium nitrate treatment showed N inorganic content (NH_4_^+^ + NO_3_^−^) around 600 mg kg^−1^, which was lower than the 1000 mg kg^−1^ of N in the 10 cm soil layer found in the UR treatment (Fig. 2). Afterwards the N content in soil decreased exponentially; during this time, nitrification inhibitors in the UR+DCD and UR+DMPP treatments maintained soil N mostly in the NH_4_^+^ form (Supplementary Fig. S1). At the 82^th^ day, soil treated with PSCU showed N content higher than other treatments, near 200 mg kg^−1^ of N, as opposed to 100 mg kg^−1^ from the UR treatment.

The original soil pH (control treatment) was around 5.1 but increased to 8 in the UR, UR+DCD and UR+DMPP treatments by seven days after fertilizer application, because of urea hydrolysis. PSCU showed pH value 1.4 units lower (pH 6.6) but treatments with calcium nitrate did not affect soil pH. In the 42^th^ day, the soil pH of the UR+DCD and UR+DMPP treatments had dropped to values around 7 (Fig. 2).

### Nitrogen cycle gene abundances

**Figure 4:**
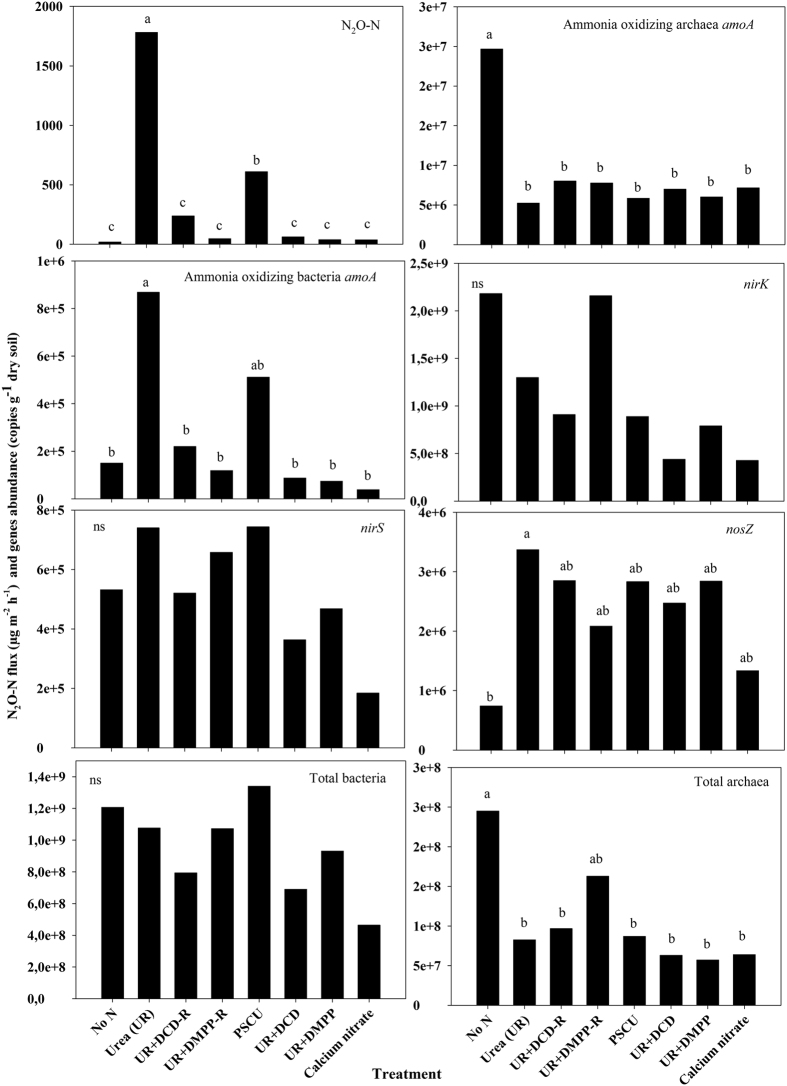
Nitrous oxide fluxes, nitrogen cycle genes (*amoA* bacteria, *amoA* archaea, *nirK*, *nirS*, *nosZ*) abundances and total bacteria and total archaea abundances in the Red Latosol soil 16 days after fertilizer application of urea with or without nitrification inhibitors (DCD and DMPP); polymer sulphur coated urea (PSCU) or calcium nitrate applied to sugarcane. R: reapplication of inhibitors in the same plots during the previous two cycles of the experiment. Means followed by the same letter did not differ (Tukey test 5%). ns: no significant.

The abundances of N cycling genes related to N_2_O emissions are depicted in Fig. 4 for one timepoint sampling that featured high N_2_O emissions: 16 days after fertilizer application, corresponding to the second soil sampling. The qPCR results from all data sampling timepoints are available in Supplementary Table S1. The abundance of *amoA* belonging to ammonia-oxidizing archaea (AOA) was lower in treatments with N sources than in the control plot, and did not show significant differences among the N sources across nearly all data sampling points. The correlation between AOA *amoA* abundance and N_2_O emissions was negative (Supplementary Table S2). The gene abundance representing total archaea showed a similar pattern as the AOA *amoA* abundance (Fig. 4 and Supplementary Table S1). On the other hand, the *amoA* abundance of ammonia-oxidizing bacteria (AOB) was best correlated with N_2_O emissions, showing a coefficient (R^2^) of 0.18 (p ≤ 0.05) (Supplementary Table S2). Over almost all data sampling points, AOB *amoA* abundances were higher in the UR treatment than in other treatments, following the data from N_2_O emissions (Fig. 4 and Supplementary Table S1). For example, concurrent to high N_2_O emissions at day 16 after fertilizer application, the coefficient of correlation (R^2^) between N_2_O and AOB *amoA* was 0.53 (Supplementary Fig. S2).

The denitrification genes *nirS* and *nosZ* as well as the 16S rRNA gene of total bacteria did not show differences in abundance between treatments over almost all data samplings. The *nirK* occurring in both ammonia-oxidizing and denitrification microorganisms had a negative correlation with N_2_O emissions, while the total bacteria abundance resulted in a positive correlation with this emission (Supplementary Table S2). In some data samplings, the abundance of the *nosZ* was higher in treatment with N than the control treatment, but no differences in *nosZ* abundance were observed between the N sources treatments (Fig. 4 and Supplementary Table S1).

A good fit, with an R^2^ of 0.47, was obtained by stepwise regression model relating N_2_O emissions to environmental variables, including the AOB *amoA* abundance, rain amount accumulated one week before GHGs measurement, NH_4_^+^-N and NO_3_-N contents, total bacteria abundance, pH, and CO_2_ emission (Supplementary Table S3). Removing the treatments without nitrification inhibitors, NH_4_^+^-N content in soil was correlated with N_2_O, while NO_3_^−^-N was not (Supplementary Tables S2 and S3).

### Bacterial community composition and diversity

Because the AOB *AmoA* abundance was correlated with N_2_O emissions, we sequenced the 16S rRNA genes from our samples to ascertain the effect of the treatments on the entire microbial (bacterial and archaeal) community. After processing the 16S rRNA amplicon sequences, the 177 samples (8 treatments × 8 timepoints × 3 replicates, excluding undersampled samples and outliers) contained between 2,000 and 56,638 sequences, with a total of 3,607,143 sequences distributed into 9,267 Operational Taxonomic Units (OTUs). Rarefaction curves indicated that most of the community diversity was captured with our sequencing depth (Supplementary Fig. S3). The top nine bacterial phyla across the samples were *Acidobacteria*, *Actinobacteria*, *Bacteroidetes*, *Firmicutes*, *Gemmatimonadetes*, *Proteobacteria* and *Verrucomicrobia* (Supplementary Table S4). The top phyla that differed between the treatments within at least one timepoint were *Firmicutes*, *Bacteroidetes*, *Nitrospira*, *Proteobacteria*, *Verrucomicrobia* and *Acidobacteria*. Shannon diversity indices of the bacterial communities ranged between 5.3 and 6.3 in treatments over all time points, and were significantly different between treatments only for days 27 (Control *versus* UR) and 82 (PSCU *versus* UR+DMPP; Table 2).

Based on phylum-level relative abundances, the samples were significantly grouped by treatment on days 7, 27, 35, 42, 82 and 158 (permutation test, P < 0.100; Table 2) with a range of observed values between 0.83 and 0.44. At day 7, three separate clusters were formed respectively with the Control and calcium nitrate treatments, the PSCU treatment and the other treatments (Supplementary Table S5). However, this clustering pattern was not observed during the remaining other days, indicating no differences in the bacterial communities between treatments. When bacterial community compositions were compared at the taxonomical level of genera, the samples were significantly grouped by treatment and the grouping was characterized by low observation values for all days except day 16 (P = 0.119; Table 2 and Supplementary Table S5). The sample treatment grouping pattern from day 7 was seen in the genus-level comparisons (Supplementary Table S5).

## Discussion

Nitrous oxide emissions from the urea treatment were higher than the emissions found in two previous sugarcane cycles in this experimental area^16^. Here, the emission factor was 1.7% of N applied, which is greater than the emission factors found in other studies of sugarcane soils in Brazil, around 0.7–1% of N applied as urea^13^,^16^,^21^. The sugarcane plant phenology may give insight into higher emissions. The N fertilizer treatments were applied 20 days after the previous sugarcane ratoon was harvested. At the time of fertilization, the soil was relatively dry – below 30% of the WFPS (Fig. 1) and sugarcane plants were still beginning to sprout. At this stage the root system was being reformed and nutrient uptake was slow, as the high soil N concentration indicated (Fig. 2). This probably led to the greater N_2_O emissions than expected. The high peaks of N_2_O emission occurred after two high rain events in the first 35 days (total 65 mm and 90 mm) on a mostly dry soil (15–20% WFPS) but with high air temperatures as shown in Fig. 1. Moreover, high correlation between N_2_O emission and accumulated rain in the week was found (Supplementary Table S2). Thus, the climatic conditions in this season contributed to the high N_2_O emission values.

A strong reduction in N_2_O emissions due to the addition of nitrification inhibitors to urea was found, as well as a lack of beneficial effects of the controlled release fertilizer PSCU, supporting similar observations in the same area^16^. The N_2_O emissions from PSCU were lower than those from UR in the first 30 days after N application, as expected from a slow-release fertilizer (Fig. 2). There was a dry spell from mid-January to the end of March (Fig. 1), which may have slowed down the release of N from PSCU. Subsequent release of N from the PSCU pellets likely led to the observed increase in N_2_O emissions (Fig. 1). In this way, the N_2_O emission from PSCU had lower peaks than those of UR but lasted longer (Fig. 3). Thus, in the end of the experiment cumulated N_2_O of UR and PSCU emissions were similar, suggesting that PSCU is not an environmentally friendly N source during one cycle of sugarcane.

In the present study, the calcium nitrate treatment showed very small N_2_O emissions that were similar to those of the control plots or plots with urea and nitrification inhibitors (NIs). In the present study intensive GHG measurements under field condition were performed over a whole yearly cycle of sugarcane. We maintain that this is the first field study demonstrating much lower N_2_O emissions of a nitrate-N source in comparison to high emissions with urea-N or NH_4_^+^-N sources; the reduction in N_2_O emissions were 98% when compared to urea. This reduction in emissions might be attributed to the high drainage capacity of the soil of the present study, which was classified as Typic Hapludox^22^ or Red Latosol^10^. Water accumulation does not tend to occur in these soil profiles, and consequently, favourable conditions for denitrifiers are avoided.

Under controlled conditions with ^15^N-labeled sources, denitrification was more important at the high soil moisture (75% WFPS), while N_2_O emissions with NH_4_^+^ fertilizers were two times higher than with NO_3_^−^ fertilization at 60% WFPS^7^. In our study WFPS reached a maximum of 40% (Fig. 1), which is more favourable for nitrification. The O_2_ concentrations were likely not low enough to favour the denitrification process in relation to nitrification.

An alternate explanation for the low N_2_O emissions in the calcium nitrate treatment could be NO_3_^−^ leaching. Indeed, the N concentration in the 0–10 cm soil layer of the calcium nitrate treatment was lower than that observed with the other N sources (Fig. 2). However, with only 70 mm of rain on a dry soil in 15 days, nitrate is unlikely to have moved beyond 30 cm. Moreover studies with ^15^N labelling showed little NO_3_^−^ leaching in sugarcane fields in Brazil^23^,^24^. Therefore, NO_3_^−^ leaching was not expected to explain the small amount of N_2_O emission found with calcium nitrate in the present study. However, further studies should include NO_3_^−^ leaching measurements to confirm the present data.

Another aspect that may have contributed to the small N_2_O emission in the calcium nitrate treatment was the relatively low organic carbon content in the soil, approximately 1%^16^. Sugarcane trash and vinasse have been reported to increase N_2_O emissions^25^, especially under high soil moisture conditions^25^. Here, we did not include in our treatments C sources such as vinasse, filter cake or sugarcane trash, common sugarcane residues or by-products. These residues can favour not only denitrifiers but also nitrifiers and other microorganisms related to the N cycle^26^. Application of exclusively NO_3_^−^-N sources with the addition of C sources, as commonly applied during sugarcane production, may result in N_2_O emissions different from those observed here and deserves further attention.

Smaller N_2_O emissions from calcium nitrate as compared to UR or NH_4_-based fertilizer have been previously reported^27^,^28^. However, in one study, N_2_O emissions observed with all the studied N sources were low (around 0.5% of the N applied), which makes it difficult to compare the treatments^28^. In a field grown with maize in Brazil, no differences were reported between UR and calcium nitrate in N_2_O emissions, but the emission factor was 0.2% of the N applied^11^. In our study, the N_2_O emissions from UR were high at 1.7% of N applied; the low N_2_O emission under calcium nitrate occurred concurrently to high N_2_O emissions from UR treatment, which highlights the relevance of the present study.

If soil moisture conditions are favourable to denitrification, nitrate-based N fertilizers may produce higher N_2_O emissions than urea or ammonium fertilizers. That is the case of the study conducted in a Gleysol soil in which the WFPS was above 60% during most of the experimental period^29^.

Based on Between-Class ordinations of the 16S rRNA compositional data as well as the total 16S rRNA gene copy numbers, the bacterial community appeared to be more affected by sampling day than by treatment. This suggests overall a minimal impact of the treatments on bacterial community composition and diversity. Though further work should examine the long-term impacts, there appears to be a low short-term impact of NIs on the bacterial community. Culturing or shotgun metagenome and metatranscriptome techniques may provide future avenues to illuminate the activity of specific nitrifiers under the environmental conditions in this study and to enhance predictions of N_2_O emissions due to nitrification in tropical soils.

Archaeal *amoA* abundances were highest in the control treatment compared to the treatments with any N sources. Elevated ammonia concentrations and higher soil pH are suggested to favour bacteria compared to archaea^8^,^18^,^30^. Interestingly, the plots with calcium nitrate also showed a reduction in archaeal *amoA* abundance compared to the control. This may reflect the accumulated effect of ammonium nitrate applied in the two previous cycles as this was the N source previously used in a separate study^16^.

Significant correlations between N_2_O emissions and *amoA* abundances were found for ammonia-oxidizing bacteria (AOB), indicating that in our study, N_2_O emissions occurred via nitrification. During the first month after UR application, a peak in the AOB *amoA* abundances was observed. Concurrently, the nitrate content in soil increased whereas ammonium decreased, soil pH decreased from 8 to around 6.3, and the soil temperature was 25 °C (Supplementary Fig. S5). Thus, the soil and climatic conditions were favourable to AOB and N_2_O emissions from nitrification.

Apart from identifying nitrification as the likely source of N_2_O emissions here, the data also suggest that denitrification was very low. No differences among treatments nor significant correlations between N_2_O emissions and the genes encoding denitrification process as *nirK*, *nirS* and *nosZ* abundances were observed. Further, the model that best estimated N_2_O emissions included bacterial *amoA* abundances and N present in the NH_4_^+^ form but not in NO_3_^−^. Thus, our gene abundance data supported the results of the low N_2_O emission data obtained from application of nitrate as the N source.

Significant correlation between AOB *amoA* abundance and N_2_O emissions was also shown. Under controlled conditions, Venterea *et al*.^31^ found a high correlation of N_2_O emissions from urea and NO_2_^−^-N content in soil resulting from increased bacterial *amoA* abundance with no increase in the abundance of the *nxr* gene, which encodes for nitrite oxidation. The authors discussed that N_2_O emissions occurred more during nitrification than denitrification, similar to the results found here. Dicyandiamide (DCD) application with cattle urine effectively inhibited the growth of AOB and reduced N_2_O emissions as well as the numbers of the *nirK* gene, which encodes for a nitrate reductase^8^. Since DCD did not affect the abundance of other denitrification genes, the authors concluded that AOB, including nitrifier denitrifiers containing *nirK*, were the main contributors to N_2_O emissions^8^. In the present study no evidences relating *nirK* and N_2_O emissions was found, but the nitrifier denitrification process could have great contribution to N_2_O released due the presence of the gene *norB* in AOB^4^,^5^,^32^. Besides nitrifier denitrification, nitrous oxide could be emitted during oxidation of hydroxylamine by ammonia-oxidizing bacteria^33^, heterotrophic nitrifiers^34^,^35^, and/or abiotic chemodenitrification^36^. Abiotic N_2_O emissions also occur due nitrite reduction by organic and inorganic compounds as amine, copper and iron^4^,^37^. Others processes that could be involved in N_2_O emissions are abiotic or biotic co-denitrification, by archaea, bacteria or fungi. In co-denitrification, a reducing compound as NO^−^, NO_2_^−^ or NO_3_^−^ combined with organic N, hydroxylamine or ammonium generates N_2_O emissions in oxic and anoxic conditions^6^,^36^. More studies targeting these reactions can pin down the relative contribution of factors explaining N_2_O fluxes from nitrification. The present study showed high N_2_O losses from urea, but very small from a nitrate fertilizer source and nitrification was the most relevant microbial process associated with such losses, which has not been reported in soil with sugarcane. The relationship between N_2_O emissions and bacterial *amoA* abundances may, therefore be a useful indicator for N management strategies to mitigate N_2_O emissions in tropical soils. Other classes of soils and N sources are necessary to confirm our data.

## Methods

### Experimental set up

The present experiment was carried out in the 2013/14 season, corresponding to the third ratoon cycle of sugarcane, the variety SP791011, in the experimental area of the Agronomic Institute in Campinas, Brazil (22°52′15″ S, 47°04′57″ W). The soil in the area was classified as Typic Hapludox or Red Latosol^10^,^22^. The same experiment was carried out during the seasons of 2011/12 and 2012/13^16^. However, in the 2013/14 season an extra treatment with calcium nitrate was included to consider N_2_O emissions due to nitrification or denitrification processes. Here, soil samples were collected in order to associate greenhouse gases (GHG) emissions with the microbial processes that were involved. The treatments were: 1) Control plot without N fertilization (control); 2) urea (UR); 3) UR + DCD; 4) UR + DMPP; 5) Polymer and Sulphur Coated Urea (PSCU); 6) UR + DCD-R; 7) UR + DMPP-R; 8) Calcium Nitrate. R stands for reapplication of inhibitors in the same plots during the previous two cycles of the experiment. The fertilizers were applied on 19 December 2013, 20 days after the harvest of the previous cycle. Phosphorus and potassium were concurrently applied to all plots at rates of 20 and 100 kg ha^−1^ of P and K, respectively.

Nitrogen was applied at a rate of 120 kg ha^−1^; the nitrification inhibitor DCD (Sigma Aldrich) was added in a dose of 5% DCD-N in relation to urea-N whereas DMPP (powder form) was added as 1% DMPP (w/w) to urea-N; PSCU was produced by Produquímica (Produquímica Ltda, Brazil) and calcium nitrate by Yara (Yara International ASA). Fertilizers were incorporated at a 5 cm soil depth to avoid NH_3_ volatilization from urea and the effect of NIs on this N loss^38^. The fertilizers were applied on either side of the plant row, 10 cm away from the recently harvested sugarcane plants. On one side of the plant row the greenhouse gases were measured; on the opposite side of the same plant, soil for chemical and molecular microbial analyses was collected.

Sugarcane yields were not measured in this study because the amount of N lost as N_2_O is generally much too low to affect yields. Furthermore, the plot size necessary to evaluate yields usually exceeds 100 m^2^. Because our focus was on GHGs emissions, which are dependent on localized soil conditions, small plots were chosen. In our study, large plots were not only unnecessary but would contribute to noise in the gas flux data.

### Greenhouse gases analysis

Greenhouse gases were collected using static chambers^16^. Chambers were fixed in the soil 5 cm deep along two 25-m long rows of sugarcane. In total, 32 chambers were used, with four replicates per treatment, in a completely randomised design. Gases were sampled in the morning and three times per week during the first three months after fertilizer application, then biweekly as previously done^16^. In each sampling date, gas samples were taken at three time intervals: 1, 15, and 30 minutes.

After sampling, the gases were immediately stored in pre-evacuated Extainers vials (Labco Limited, Ceredigion, United Kingdom) and analysed in a Shimadzu gas chromatograph (GC-2014). Cumulated gas emissions were calculated by linear interpolation between gas samplings periods. Details of the procedures used for gas analysis and calculations are described elsewhere^16^,^21^.

### Soil chemical analysis

Soil samples (0–10 cm depth) were collected more intensively in the first two months after fertilizer application, a period corresponding to higher N_2_O emissions. Using an auger, three subsamples were collected as a composite sample per experimental plot. In total, eight soil sampling campaigns were collected at 7, 16, 18, 27, 35, 42, 82 and 158 days after fertilizer application. The soil samples were stored in plastic bags at −20 °C. Gravimetric moisture after constant weight was attained at 105 °C. The water-filled pore space (WFPS) was calculated considering soil bulk density and porosity determined at the beginning of the experiment. Soil pH was measured in CaCl_2_ (0.0125mol L^−1^) and NH_4_^+^-N and NO_3_^−^-N contents were determined by steam distillation after soil extraction in 2 mol L^−1^ KCl solution^39^.

### Real-time PCR analysis

Soil subsamples (20 g) were stored at −80 °C for molecular analyses. Total soil DNA was extracted from 0.25 g of soil using the Power Soil kit (Mobio, Carlsbad, CA USA) following the manufacturer’s instructions. The quantity and quality of DNA was measured by NanoDrop ND-1000 spectrophotometer (NanoDrop Technologies, Montchanin, USA). The DNA samples were diluted in water free of DNase and RNase (Sigma Aldrich) and the abundance of the genes encoding for nitrification and for denitrification processes were quantified by quantitative real-time PCR with a Qiagen Rotor-Gene Q6000 cycler (RO212226). A reaction was performed in total volume of 12 μl, containing 6 μl Sybrgreen Bioline SensiFAST SYBR non-rox mix, 0.5 μl of each primer (5 pmol) and 5 μl of DNA (3 ng). Exceptions were the reaction for the *nirK* amplification, for which the Sybrgreen Qiagen Rotor-Gene SYBR Green PCR Kit was used, and the *nosZ* amplification, for which the starting DNA concentration was 30 ng. Reactions were performed by a QIAgility robot (003516).

The thermal conditions of each gene amplification are listed in Supplementary Table S5. Acquisition was done at 72 °C (cycle A) or 82–86 °C (cycle B) to avoid primer dimers. Melt curve analysis was done at 55–99 °C to confirm specificity; the qPCR products were checked by agarose gel electrophoresis to confirm the desired amplicon size. Plasmid DNA from microorganisms containing the gene of interest or from environmental samples were used for the standard curve and then cloned into vectors as described in Table S5. Normal PCR reactions were carried out with similar thermal conditions as qPCR to confirm the fragment size of interest, then cloned and transformed into JM109 High Efficiency Competent Cells (Promega, *In Vitro* Technologies, Auckland, New Zealand). After overnight bacterial growth in LB medium with ampicillin at 37 °C, plasmids were extracted using the PureLin Quick Plasmid Miniprep Kit (Life Technologies, Auckland, New Zealand). The quantity and quality of plasmid DNA were checked by spectrophotometer (NanoDrop ND-1000 Technologies, Montchanin, USA). Standard dilutions were obtained from 10 to 10^8^ copies/μl of each gene. Each run included a DNA template, standard, and a no-DNA control – water free of DNase and RNase (Sigma Aldrich) – done in duplicate. Reaction efficiency was 89–105% and R^2^ values ranged from 0.94 to 0.99.

### 16S rRNA partial gene sequencing

To assess the impact of the treatments on the bacterial community, we sequenced the 16S rRNA gene marker from total DNA extracted from the soil samples. The V4 region of the 16S rRNA gene was amplified by using archaeal/bacterial primers 515F (5′-GTGCCAGCMGCCGCGGTAA-3′) and 806R (5′-GGACTACVSGGGTATCTAAT-3′). The samples were PCR-amplified using barcoded primers linked with the Ion adapter “A” sequence (5′-CCATCTCATCCCTGCGTGTCTCCGACTCAG-3′) and Ion adapter “P1” sequence (5′-CCTCTCTATGGGCAGTCGGTGAT-3′) to obtain a sequence of primer composed for A-barcode-806R and P1-515F adapter and primers. The 16S rRNA gene amplifications for library preparation were performed on the C1000 thermocycler (Biorad, Hercules, CA, USA) with thermal conditions of 95 °C-5 min.; 35× 95 °C-30 s, 53 °C-30 s, 72 °C-60 s; 72 °C-10 min. A reaction of 25 μl in total was done, including 2.5 μl of 10X PCR Buffer, 2.5 μl dNTPs (200 μM), 0.25 μl of each primer (0.1 pmol/μl), 0.2 μl of fast startExp-Polymerase (0.056 U) and 1 μl of DNA (0.6 ng). The reactions were carried out in duplicate and included a negative control. The amplicons were checked by gel electrophoresis. The PCR products were purified by Agencourt AMPURE XP to remove primer dimers, then quantified by Quant-iT PicoGreen and equimolar mixed for sequencing using the PGM Ion Torrent (Life Technologies).

### 16S rRNA amplicon sequences processing

MOTHUR Version 1.34.2 was used to process the 16S rRNA partial genes sequences, implemented using a Snakemake workflow on a 32-node server running Linux Ubuntu 14.4^40^. Forward and reverse primer sequences were removed from each sample FASTQ file using Flexbar version 2.5^41^. Reads were filtered based on sequence quality by running the Sickle tool (minimum quality score 25, minimum length 150). Filtered reads were converted to FASTA format and concatenated into a single file, then clustered into OTUs using the UPARSE strategy of dereplication, sorting by abundance with at least two sequences and clustering using the UCLUST smallmem algorithm^42^. These steps were performed with VSEARCH version 1.0.10, which is an open-source and 64-bit multithreaded compatible alternative to USEARCH. Chimeric sequences were detected using the UCHIME algorithm^43^ implemented in VSEARCH. All reads before the dereplication step were mapped to OTUs using the USEARCH_global method implemented in VSEARCH to create an OTU table and then converted to the BIOM-Format 1.3.1^44^. Last, taxonomic information for each OTU was added to the BIOM file using the RDP Classifier version 2.10^45^.

### Statistical analysis – gas fluxes and gene abundances

Daily GHG fluxes, cumulated emissions of N_2_O, CO_2_, CH_4_ and gene abundance values were checked for normal distribution of residues by Shapiro-Wilk test, and then submitted to variance analysis (ANOVA) and the means compared by Tukey’s test at P ≤ 0.05. Soil pH was transformed to H^+^: 10^−pH^ before statistical analysis. Linear correlations between N_2_O fluxes and environmental variables were evaluated at the 5% level of significance. Multiple linear regressions, which were selected by the stepwise process at p ≤ 0.05, were fitted between N_2_O fluxes and environmental variables. When necessary, the N_2_O flux values were log(x) transformed and rechecked to obtain a normal distribution of residues and variance stability^46^. The calculations were performed with the SISVAR statistical software^47^ and graphics plotted using Sigma Plot^48^.

Cumulative N_2_O emissions as a function of time were fitted by sigmoidal or exponential equations, for which the sigmoid equation was:


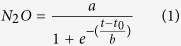


where *N*_*2*_*O* is the cumulative N_2_O-N emission, g ha^−1^, *t* is the time in days after fertilizer application, and *a*, *t*_*0*_ and *b* are equation parameters, where *a* is the maximum loss and *t*_*0*_ is the time in which 50% of maximum loss occurs.

The exponential rise to the maximum model had the following equation:


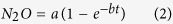


where *N*_*2*_*O* is the cumulative N_2_O-N emission, g ha^−1^, *t* is the time in days after fertilizer application, and *a* and *b* are equation parameters, where *a* is the maximum loss and *b* is the rate of rise.

### Statistical analysis – 16S rRNA amplicon sequence data

The 16S rRNA samples were analysed to compare bacterial community alpha diversity and composition across treatments and time point (days 7, 16, 18, 27, 35, 42, 82 and 158). The BIOM files were handled with the “phyloseq” package^49^ in R^50^. Rarefaction curves were generated to ensure adequate sequencing depth across samples. Discarding undersequenced samples, the minimum sample size was 2000. For alpha diversity analyses, the 16S rRNA samples were rarefied to 2000 sequences using the “vegan” package^51^. Renyi diversities at alpha level 1, corresponding to the Shannon diversity index were kept (“BiodiversityR” R package). The Shannon diversity data was furthermore subjected to Kruskal-Wallis tests among treatments and the Kruskal-Wallis multiple comparison test between treatments using the “pgirmess” R package.

Comparisons of bacterial community compositions were evaluated using the Statistical Analysis of Metagenomic Profiles (STAMP) software^52^. The top nine Bacterial phyla based on relative abundances across all samples were compared among and between treatments for each time point. The unclassified sequences were removed prior to analysis. The ANOVA statistical and Tukey-Kramer post-hoc tests (CI 95%) were applied using the Benjamini Hochberg multiple test correction. To explore beta diversity (treatment differences) of the bacterial communities, Between-Class Analysis (BCA) of the non-rarefied 16S rRNA samples grouped by treatment was performed using the “ade4” R package^53^. First, with unclassified sequences removed, correspondence analyses of the compositional data agglomerated at the rank of Phylum and Genus were conducted, followed by BCA. Further, the BCA groups for the phyla and genera analyses were tested using the Monte-Carlo permutation method with 999 repetitions.

## Additional Information

**Accession codes:** European Nucleotide Archive study accession number PRJEB13027.

**How to cite this article**: Soares, J. R. *et al*. Nitrous oxide emission related to ammonia-oxidizing bacteria and mitigation options from N fertilization in a tropical soil. *Sci. Rep.* **6**, 30349; doi: 10.1038/srep30349 (2016).

## Supplementary Material

Supplementary Information

## Figures and Tables

**Table 1:** Cumulated nitrous oxide, carbon dioxide and methane emissions from Red Latosol soil during 278 days after application of urea with or without nitrification inhibitors (DCD and DMPP), polymer sulphur coated urea (PSCU) and calcium nitrate applied to sugarcane.

Treatments	N_2_O-N					CO_2_-C		CH_4_-C	
	g ha^−1^	log*		% N applied†	Reduction (%)	kg ha^−1^*		g ha^−1^*	
Control	286	2.4	C	—	—	5835	ns	−598	ns
UR	2301	3.4	A	1.68	—	5933		−633	
UR+DCD-R	531	2.7	B	0.20	88	5883		−612	
UR+DMPP-R	350	2.5	C	0.05	97	5871		−532	
PSCU	2165	3.3	A	1.57	7	5912		−648	
UR+DCD	410	2.6	BC	0.10	94	5859		−633	
UR+DMPP	353	2.5	C	0.06	97	5897		−656	
Calcium nitrate	329	2.5	C	0.04	98	5973		−600	
*P* value		<0.00001				0.9769		0.9328	

^*^Tukey test, p ≤ 0.05; ns: no significant; N_2_O-N: g ha^−1^ transformed in log(X)

^†^Results from treatment without N were subtracted for this calculation. —R means reapplication of inhibitors in the same plot in the two preceding years. Different characteristics in the column of N_2_O mean significant differences (p < 0.05) between the values.

**Table 2:** Shannon indices and Between-Class Analysis (BCA) ordinations of the bacterial communities present in the Red Latosol soil under treatments with urea with or without nitrification inhibitors (DCD and DMPP); polymer sulphur coated urea (PSCU) or calcium nitrate applied to sugarcane.

Treatments	16S rRNA gene diversity (Shannon index)															
	7 DAF*		16		18		27		35		42		82		158	
Control	6.2	ns	6.1	ns	6.0	ns	6.2	a	6.1	ns	6.2	ns	6.0	ab	6.1	ns
UR	5.8		6.0		6.0		5.3	b	5.7		5.8		5.6	ab	6.0	
UR+DCD-R†	5.9		6.2		5.8		6.0	ab	6.0		6.1		5.8	ab	6.2	
UR+DMPP-R	5.6		6.1		5.9		5.8	ab	6.1		6.2		6.1	ab	6.2	
PSCU	5.9		5.9		5.8		5.8	ab	5.1		5.2		5.4	a	5.8	
UR+DCD	5.7		6.1		5.7		5.8	ab	5.9		6.0		6.0	ab	6.2	
UR+DMPP	5.7		6.0		5.9		5.8	ab	5.5		6.1		6.1	b	6.1	
Calcium nitrate	6.3		6.0		5.7		5.9	ab	5.9		6.2		6.0	ab	6.2	

^*^DAF: Days after fertilizer application. Means in column followed by same letter did not differ; ns: no significant.

^†^R means reapplication of inhibitors in same plots.
