# Supplementary material for: Nitrous oxide emission related to ammonia-oxidizing bacteria and mitigation options from N fertilization in a tropical soil
Source: Sci Rep. 2016 Jul 27;6:30349. doi: 10.1038/srep30349 (PMC4962081; doi:10.1038/srep30349)
Supplement: Supplementary Information [file srep30349-s1.pdf]

Nitrous oxide emission related to ammonia-oxidizing bacteria and mitigation options from N fertilization in a tropical soil

Johnny R. Soares[1,2], Noriko A. Cassman[1], Anna M. Kielak[1], Agata Pijl[1], Janaína B. Carmo[3], Kesia S. Lourenço[1,2], Hendrikus J. Laanbroek[1,4], Heitor Cantarella[2] & Eiko E. Kuramae[1,*]

[1] Department of Microbial Ecology, Netherlands Institute of Ecology, 6708 PB, Wageningen, Netherlands.

[2] Soils and Environmental Resources Center, Agronomic Institute of Campinas, P.O. Box 28, 13012-970, Campinas, SP, Brazil.

[3] Environmental Science Department, Federal University of São Carlos, 1852-780, Sorocaba, SP, Brazil.

[4] Institute of Environmental Biology, Utrecht University, Netherlands

[*]Equal contribution

[§]Corresponding author: e.kuramae@nioo.knaw.nl

Supplementary Material

Table S1. Nitrous oxide fluxes, nitrogen cycle genes abundance and total bacterial and total archaea abundance in the Red Latosol soil as affected by treatments with urea with or without nitrification inhibitors (DCD and DMPP); polymer sulfur coated urea (PSCU) or calcium nitrate applied to sugarcane.

Treatment	N_2O-N	AOA *amoA*	AOB *amoA*	*nirK*	*nirS*	*nosZ*	Total archaea	Toral bacteria
7 DAF†	µg m^{-2} h^{-1}	10^6 copies g^{-1}	10^5 copies g^{-1}	10^8 copies g^{-1}	10^5 copies g^{-1}	10^6 copies g^{-1}	10^7 copies g^{-1}	10^9 copies g^{-1}
Control	3.8 c	7.5 a	2.3 b	7.8 ns	4.2 ns	15.9 ns	9.7 ns	2.6 abc
UR	82.0 a	2.0 b	12.2 a	6.8	4.6	21.3	4.5	5.9 a
UR+DCD - R‡	12.9 c	3.5 b	2.8 b	7.1	3.9	13.9	5.1	4.9 abc
UR+DMPP - R	5.8 c	3.2 b	3.0 b	8.5	4.8	18.7	8.6	5.6 ab
PSCU	55.5 b	1.9 b	2.6 b	4.0	1.4	6.7	2.9	1.3 bc
UR+DCD	8.9 c	3.5 b	0.6 b	2.0	0.8	5.3	2.3	1.2 c
UR+DMPP	8.3 c	2.5 b	1.4 b	3.7	2.7	10.2	3.1	2.0 abc
Calcium nitrate	12.8 c	1.6 b	0.5 b	1.1	1.7	3.7	1.5	0.8 c
16 DAF								
Control	18.7 c	41.1 a	1.5 b	2.2 ns	5.3 ns	0.7 b	24.5 a	1.2 ns
UR	1781.3 a	8.7 b	8.7 a	1.3	7.4	3.4 a	8.3 b	1.1
UR+DCD - R	238.4 c	13.4 b	2.2 b	0.9	5.2	2.9 ab	9.7 b	0.8
UR+DMPP - R	47.7 c	12.9 b	1.2 b	2.2	6.6	2.1 ab	16.3 ab	1.1
PSCU	609.8 b	9.8 b	5.1 ab	0.9	7.4	2.8 ab	8.7 b	1.3
UR+DCD	62.2 c	11.7 b	0.9 b	0.4	3.6	2.5 ab	6.3 b	0.7
UR+DMPP	39.3 c	10.0 b	0.7 b	0.8	4.7	2.8 ab	5.7 b	0.9
Calcium nitrate	36.9 c	11.9 b	0.4 b	0.4	1.8	1.3 ab	6.4 b	0.5
18 DAF								
Control	15.3 c	23.6 a	2.8 b	14.5 ns	35.2 ns	0.7 b	18.0 a	1.0 ns
UR	1187.0 a	8.5 b	10.7 ab	7.9	14.2	1.6 a	3.2 b	0.7
UR+DCD - R	110.6 c	4.3 b	16.7 a	10.0	20.0	1.0 ab	5.1 b	1.1
UR+DMPP - R	27.6 c	6.4 b	1.7 b	9.3	27.7	1.2 ab	5.2 b	1.0
PSCU	558.0 b	2.2 b	6.6 ab	2.8	6.0	0.8 b	2.0 b	0.3
UR+DCD	53.8 c	7.4 b	4.4 b	5.8	9.6	1.1 ab	7.3 b	0.9
UR+DMPP	20.9 c	3.5 b	1.9 b	5.9	6.4	1.0 ab	4.7 b	0.5
Calcium nitrate	27.4 c	9.0 b	1.1 b	4.1	10.7	0.5 b	9.9 ab	0.5

Table S1. Continued.

Treatment	N_2O-N	AOA *amoA*	AOB *amoA*	*nirK*	*nirS*	*nosZ*	Total archaea	Total bacteria
27 DAF	μg m^{-2} h^{-1}	10^6 copies g^{-1}	10^5 copies g^{-1}	10^8 copies g^{-1}	10^5 copies g^{-1}	10^6 copies g^{-1}	10^7 copies g^{-1}	10^9 copies g^{-1}
Control	8.1 c	27.8 a	6.8 bc	15.4 a	5.3 a	1.8 abc	14.4 a	1.3 ns
UR	1356.3 a	2.1 bc	53.8 a	7.3 ab	4.1 ab	2.2 a	2.0 c	0.9
UR+DCD - R	163.8 c	6.0 bc	31.3 ab	7.3 ab	4.9 ab	1.7 abc	4.1 bc	0.7
UR+DMPP - R	10.7 c	8.3 b	3.7 c	11.1 ab	6.5 a	2.1 ab	8.3 ab	1.4
PSCU	704.8 b	1.5 c	17.9 bc	3.9 b	2.1 b	1.3 bc	2.4 bc	0.8
UR+DCD	77.3 c	5.0 bc	10.2 bc	5.2 ab	2.2 b	1.4 abc	3.2 bc	0.8
UR+DMPP	39.3 c	6.0 bc	4.4 bc	7.1 ab	2.6 b	1.7 abc	3.1 bc	0.9
Calcium nitrate	36.9 c	6.8 bc	1.8 c	3.6 b	0.8 b	0.9 c	3.0 bc	0.4
35 DAF								
Control	8.4 b	12.4 a	1.2 b	5.5 ns	1.2 ns	2.1 ns	3.7 ns	0.3 ns
UR	1137.0 a	4.0 ab	11.8 a	6.2	1.3	2.0	2.4	0.5
UR+DCD - R	83.9 b	4.4 ab	5.7 b	2.6	0.9	2.2	2.0	0.3
UR+DMPP - R	42.4 b	3.8 ab	1.6 b	5.6	1.3	1.3	3,0	0.5
PSCU	1167.5 a	1.4 b	2.8 b	2.5	0.3	1.2	1,0	0.2
UR+DCD	264.5 b	4.8 ab	3.4 b	3.0	0.6	1.9	1,7	0.3
UR+DMPP	105.4 b	9.1 ab	1.6 b	4.3	1.4	3.1	2,1	0.4
Calcium nitrate	28.7 b	9.4 ab	1.0 b	4.7	0.7	2.0	2,8	0.3
42 DAF								
Control	4.0 b	28.1 a	1.6 ns	13.5 ns	3.4 ns	2.5 ns	7,9 a	0.7 ns
UR	225.3 ab	1.4 b	8.5	4.5	2.0	2.4	0,8 b	0.4
UR+DCD - R	10.8 b	3.3 b	8.0	10.2	2.8	1.7	3,6 ab	0.6
UR+DMPP - R	22.1 b	5.7 b	3.0	11.9	4.2	2.1	3,5 ab	0.7
PSCU	312.9 a	1.8 b	6.5	4.0	1.6	1.7	1,0 b	0.3
UR+DCD	15.3 b	6.1 b	9.7	6.0	1.4	1.7	3,3 ab	0.5
UR+DMPP	10.7 b	5.5 b	1.6	7.7	2.4	1.6	3,3 ab	0.6
Calcium nitrate	6.5 b	7.0 b	1.2	5.0	1.0	1.1	2,7 ab	0.4

Continue in next page.

Table S1. Continued.

Treatment	N_2O-N		AOA *amoA*		AOB *amoA*		*nirK*		*nirS*		*nosZ*		Total archaea		Total bacteria	
82 DAF	$\mu g\ m^{-2}\ h^{-1}$		10^6 copies g^{-1}		10^5 copies g^{-1}		10^8 copies g^{-1}		10^5 copies g^{-1}		10^6 copies g^{-1}		10^7 copies g^{-1}		10^9 copies g^{-1}	
Control	0.2	b	40.7	a	1.7	b	22.1	ns	4.6	ab	0.6	c	13.8	ns	1.3	ns
UR	1.6	b	1.0	b	10.7	a	2.9		1.1	b	1.7	abc	0.8		0.2	
UR+DCD - R	0.1	b	1.9	b	2.8	b	13.4		9.2	ab	1.1	bc	6.2		0.8	
UR+DMPP - R	0.2	b	6.2	b	0.8	b	3.0		3.1	ab	0.8	bc	4.0		0.1	
PSCU	8.8	a	1.4	b	4.8	ab	1.9		1.4	b	0.7	c	1.0		0.1	
UR+DCD	0.2	b	22.4	ab	3.7	b	19.5		8.8	ab	2.2	ab	9.8		2.2	
UR+DMPP	0.1	b	23.2	ab	2.4	b	29.6		13.0	ab	3.1	a	12.6		2.4	
Calcium nitrate	0.1	b	51.3	a	2.4	b	19.7		17.4	a	2.0	abc	20.8		2.6	
158 DAF																
Control	0.1	b	35.3	a	2.9	ab	23.9	ns	14.7	ns	1.0	ns	9.2	a	1.1	ns
UR	0.3	b	2.8	b	4.2	ab	6.3		5.2		1.0		2.4	ab	0.5	
UR+DCD - R	0.2	b	10.1	b	5.7	a	13.8		16.5		0.9		6.2	ab	2.0	
UR+DMPP - R	0.1	b	5.2	b	1.3	b	7.0		5.1		0.8		3.7	ab	0.5	
PSCU	4.6	a	7.7	b	4.1	ab	6.6		4.0		0.8		2.4	ab	0.7	
UR+DCD	0.1	b	6.5	b	1.7	b	5.5		4.2		0.7		0.5	b	0.5	
UR+DMPP	0.2	b	10.0	b	0.9	b	5.5		4.7		0.9		0.3	b	0.4	
Calcium nitrate	0.0	b	11.9	b	1.3	b	13.1		4.4		0.7		0.5	b	0.6	

Means followed by same letter in column per DAF did not differ by Tukey test 5%. ns: no significant. †Days after fertilizer application. ‡ R means reapplication of inhibitors in same plot. Continue in next page.

Table S2. Linear regression and coefficients (R^2) relating daily N_2O flux to environmental variables from Red Latosol soil grown with sugarcane after application of urea with or without nitrification inhibitors (DCD and DMPP), polymer sulfur coated urea (PSCU) and calcium nitrate (n = 256).

	All treatments		Without nitrification inhibitors	
Variable (x)†	Regression*	R^2	Regression	R^2
NH_4^+-N	ns	-	y = 1.87 + 0.00073x	0.0879
NO_3^--N	y = 1.64 + 0.0016x	0.0389	ns	-
WFPS	y = 0.74 + 0.031x	0.1021	y = 0.82 + 0.034x	0.0864
Temp. air	ns	-	ns	-
Temp. soil	ns	-	ns	-
pH	y = 1.70 + 2.4e4x	0.0214	ns	-
Rain day	y = 1.58 + 0.013x	0.0818	y = 1.77 + 0.013x	0.0609
Rain week	y = 1.40 + 0.0081x	0.1589	y = 1.60 + 0.0078x	0.1017
CO_2-C	ns	-	ns	-
CH_4-C	y = 1.78 + 0.0073x	0.0243	y = 2.02 + 0.011x	0.0422
AOA *amoA*	y = 1.88 - 2.83e-8x	0.0990	y = 2.18 - 3.83e-8x	0.2107
AOB *amoA*	y = 1.56 + 2.94e-7x	0.1786	y = 1.71 + 3.02e-7x	0.1944
nirK	y = 1.82 - 1.32e-11x	0.0405	y = 2.03 - 1.55e-11x	0.0485
nirS	ns	-	ns	-
nosZ	ns	-	ns	-
Total bacteria	y = 1.80 + 7.83e-11x	0.0297	ns	-
Total archaea	y = 1.84 - 2.28e-9x	0.0532	y = 2.10 - 3.24e-9x	0.1076

† N_2O-N: µg m^{-2} h^{-1} transformed in log(X+10); CO_2-C: mg m^{-2} h^{-1}; CH_4-C: µg m^{-2} h^{-1}; NH_4^+-N and NO_3^--N: mg kg^{-1} 0-10 cm soil; Rainweek and Rainday: mm, Temp. air and soil: ºC; WFPS: Water-filled pore space, %; AOB *amoA*, AOA *amoA*, *nir*K, *nir*S, *nos*Z, total bacteria and total archaea: copies gene g^{-1} dry soil; pH-$CaCl_2$: transformed in H^+, 10^{-pH}. *Regression equations significant at $p \leq 0.05$ except where otherwise indicated (ns: no significant).

Table S3. Multiple linear regression parameters (β) and coefficients (R^2) relating daily N_2O flux to environmental variables after fertilizer application to sugarcane (n = 256).

Model*	Parameters	R^2
All treatments†		
N_2O-N = β0 + βAOB + βRainweek + βpH + βNH_4^+-N + βBacteria + βCO_2 + βNO_3^--N ‡	0.84 + 2.96e-7AOB + 0.0097Rainweek + 3.3e4pH + 0.0050NH_4^+-N - 0.9e-10Bacteria + 0.0019NO_3^--N + 0.00093CO_2	0.4741
Without nitrification inhibitors		
N_2O-N = β0 + βAOB + βRainweek + βNH_4^+-N + βBacteria + βpH+ βCO_2	0.96 + 2.93e-7AOB + 0.0096Rainweek + 0.0012NH_4^+-N - 1.8e-10Bacteria + 3.0e4pH + 0.0023CO_2	0.5267

* Stepwise selection, $p \leq 0.05$. † Treatments: Control, Urea (UR), UR+DCD, UR+DMPP, polymer sulfur coated urea and calcium nitrate. ‡ N_2O-N: µg m^{-2} h^{-1} transformed in log(X+10); AOB and Bacteria: *amoA* from ammonia oxidizing bacteria and all bacteria, copies gene g^{-1} dry soil; NH_4^+-N and NO_3^--N: mg kg^{-1} 0-10 cm soil; Rainweek: mm; pH-$CaCl_2$: transformed in H^+, 10^{-pH}.

Table S4. Top-nine bacterial phyla presented in the microbial communities in the Red Latosol soil under treatments with urea with or without nitrification inhibitors (DCD and DMPP); polymer sulfur coated urea (PSCU) or calcium nitrate applied to sugarcane.

Treatment	Mean relative abundance (%) of Bacterial Phyla, or effect size(corrected p-value)								
	Proteobacteria	Firmicutes	Actinobacteria	Acidobacteria	Bacteroidetes	Verrucomicrobia	Planctomycetes	Gemmatimonadetes	Nitrospirae
7 DAF†	ns	0.73 (0.07)	ns	ns	ns	ns	ns	ns	ns
Control	24	4	20	21	3	5	2	1	1
UR	27	24	23	5	4	2	1	1	0
UR+DCD - R‡	26	26	14	12	4	3	2	1	1
UR+DMPP – R	25	28	12	12	7	2	2	1	0
PSCU	44	6	24	7	3	2	2	1	0
UR+DCD	34	20	13	8	5	3	1	1	0
UR+DMPP	23	32	16	7	4	2	1	1	0
Calcium nitrate	30	9	22	13	4	4	2	1	1
16 DAF†	ns	ns	ns	ns	0.65(0.09)	ns	ns	ns	0.63(0.09)
Control	28	10	31	11	3	3	1	1	1
UR	36	15	23	4	8	1	1	1	0
UR+DCD - R‡	30	12	19	12	6	4	3	1	0
UR+DMPP – R	36	13	20	11	4	2	2	1	0
PSCU	35	18	23	5	7	1	1	1	0
UR+DCD	32	15	22	7	7	3	2	1	0
UR+DMPP	30	11	21	10	4	5	3	1	0
Calcium nitrate	22	21	24	12	2	3	2	1	0
18 DAF†	ns	ns	ns	ns	ns	ns	ns	ns	ns
Control	25	9	24	15	3	5	2	1	1
UR	34	14	24	6	6	2	2	1	0
UR+DCD - R‡	41	13	17	2	12	1	3	1	0
UR+DMPP – R	43	10	17	5	10	2	1	2	0
PSCU	38	13	23	6	6	1	1	1	0
UR+DCD	31	20	17	5	11	2	2	1	0
UR+DMPP	34	17	24	5	5	2	1	1	0
Calcium nitrate	35	18	23	6	5	2	1	1	0
27 DAF†	ns	ns	ns	ns	ns	ns	ns	ns	ns
Control	42	3	23	8	6	2	0	2	1
UR	49	7	13	3	17	1	1	2	0
UR+DCD - R‡	49	5	16	5	7	3	1	2	0
UR+DMPP – R	48	4	12	6	13	3	1	2	0
PSCU	49	6	20	3	10	1	1	2	0
UR+DCD	48	5	16	3	14	2	1	2	0
UR+DMPP	53	6	18	3	9	1	0	1	0
Calcium nitrate	45	7	19	6	6	2	1	2	0
35 DAF†	0.71(0.02)	ns	ns	ns	ns	0.67(0.04)	ns	ns	ns
Control	32	2	15	18	6	5	1	2	1
UR	48	3	15	5	12	1	1	2	0
UR+DCD - R‡	46	5	14	8	12	2	1	2	0
UR+DMPP – R	45	6	14	7	12	3	1	2	0
PSCU	57	3	12	5	13	1	1	1	0
UR+DCD	42	5	13	8	12	2	1	3	0
UR+DMPP	43	3	11	8	18	2	1	2	0
Calcium nitrate	42	10	20	5	8	2	1	3	0

Table S4 continued.

Treatment	Mean relative abundance (%) of Bacterial Phyla, or effect size(corrected p-value)								
	Proteobacteria	Firmicutes	Actinobacteria	Acidobacteria	Bacteroidetes	Verrucomicrobia	Planctomycetes	Gemmatimonadetes	Nitrospirae
42 DAF†	0.60(0.07)	ns	ns	0.60(0.09)	0.64(0.08)	ns	ns	ns	0.74(0.02)
Control	31	2	19	17	8	6	1	1	1
UR	39	5	17	7	16	3	1	2	0
UR+DCD - R‡	43	4	15	6	15	3	1	2	1
UR+DMPP – R	37	6	19	10	11	2	2	2	0
PSCU	53	2	17	4	11	1	1	1	0
UR+DCD	40	4	18	7	13	5	1	2	0
UR+DMPP	38	4	18	11	9	4	2	2	0
Calcium nitrate	37	4	19	8	13	4	1	2	0
82 DAF†	ns	0.80(0.003)	ns	ns	0.65(0.03)	ns	ns	0.72(0.01)	0.77(0.004)
Control	37	4	13	14	9	5	1	2	1
UR	47	3	15	5	16	1	1	3	0
UR+DCD - R‡	37	22	22	2	9	1	0	1	0
UR+DMPP – R	48	15	18	5	7	1	0	1	0
PSCU	50	4	20	3	14	0	0	1	0
UR+DCD	45	3	16	8	11	3	1	3	0
UR+DMPP	42	6	18	9	10	2	1	2	1
Calcium nitrate	30	20	30	4	4	2	1	1	0
158DAF†	ns	ns	ns	ns	ns	ns	ns	ns	0.70(0.06)
Control	39	3	21	9	11	3	1	2	0
UR	47	3	15	6	12	3	1	3	0
UR+DCD - R‡	42	6	22	5	11	1	0	3	0
UR+DMPP – R	43	5	23	7	9	2	1	2	0
PSCU	47	3	20	5	9	2	1	2	0
UR+DCD	43	5	18	8	10	3	1	2	0
UR+DMPP	46	3	17	8	10	2	1	3	0
Calcium nitrate	40	5	24	5	9	2	1	3	0

Significance from Tukey-Kramer post-hoc test based on Benjamini-Hochberg corrected p-values above 0.1 from STAMP analysis. ns: not significant. †Days after fertilizer application. ‡ R means reapplication of inhibitors in same plot.

Table S5. Primers and thermocycler conditions used in gene abundance analysis by real time qPCR

Target gene	Primer	Primer Sequence	Size (bp)	Thermal profile	Reference	Source of Standard - plasmid	Vector
AOA *amoA*	Arch-amoAF	5’-STAATGGTCTG GCTTAGACG-3’	635	95°C-5 min.; 40x 95°C-30s, 55°C-45s, 72°C-45s, 82°C-15s	Francis et al.[1]	Environmental Archaea	pGEM®-T Vector Systems Promega
	Arch-amoAR	5’-GCGGCCATCC ATCTGTATGT-3’					
AOB *amoA*	amoA1F	5’-GGGGTTTCT ACTGGTGGT-3’	491	95°C-5 min.; 40x 95°C-30s, 56°C-45s, 72°C-45s, 82°C-15s	Rotthauwe et al.[2]	*Nitrosomonas europaea*	pGEM®-T Vector Systems Promega
	amoA2R	5’-CCCCTCKGSA AAGCCTTCTTC-3’					
*nos*Z	nosZ2F	5’-CGCRACGGCAA SAAGGTSMSSGT-3’	267	95°C-5 min.; 40x 95°C-15s, 60°C-15s, 72°C-30s, 82°C-15s	Henry et al.[3]	*Pseudomonas stutzeri* (M13R/F)	Dh5alpha pgemTeasy PCR4-topo vector
	nosZ2R	5’-CAKRTGCAKSG CRTGGCAGAA-3’					
*nir*K	NirK876	5'-ATYGGCGG VAYGGCGA-3'	165	95°C-5 min.; 40x 95°C-15s, 63°C-30s, 72°C-30s, 82°C-15s	Henry et al.[4]	*Paracoccus denitrificans* (DSM 413)	Dh5alpha pgemTeasy PCR4-topo vector
	NirK1040	5'-GCCTCGATCA GRTTRTGGTT-3'					
*nir*S	nirScd3aF	5'-GTSAACGTSA AGGARACSGG-3'	425	95°C-5 min.; 40x 95°C-10s, 60°C-10s, 72°C-20s, 86°C-5s	Throbäck et al.[5]	*Pseudomonas stutzeri* (M13R/F)	PCR product
	nirSR3cd	5'-GASTTCGGRT GSGTCTTGA-3'					
Total bacteria	Eub338	5'-ACTCCTACGG GAGGCAGCAG-3'	200	95°C-5 min.; 40x 95°C-5s, 53°C-10s, 72°C-20s	Fierer et al.[6]	Firmicutes	Dh5alpha pgemTeasy PCR4-topo vector
	Eub518	5'-ATTACCGC GGCTGCTGG-3'					
Total archaea	Arch1017R	5'-AGGAATTGGC GGGGGAGCAC-3’	112	95°C-10 min.; 40x 95°C-10s, 60°C-10s, 72°C-20s	Klindworth et al.[7]	Environmental Archaea	Dh5alpha pgemTeasy PCR4-topo vector
	Arch915F	5’-GGCCATGCA CCWCCTCTC-3’					

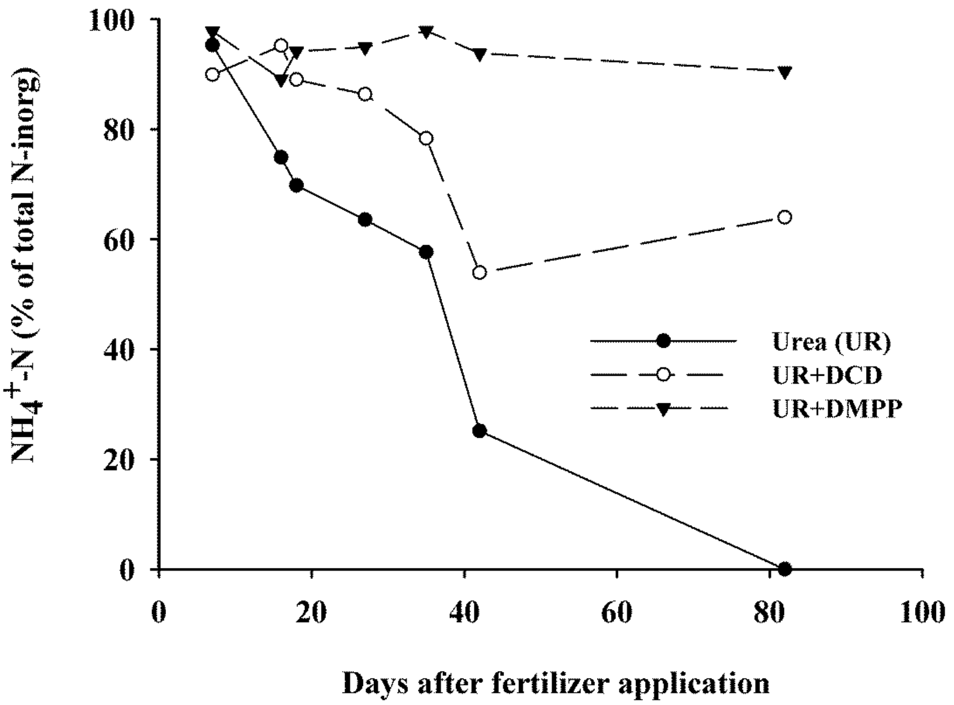

Figure S1. Percentage of NH_4^+ in relation to total inorganic N (NH_4^+ + NO_3^-) at 0 - 10 cm soil depth after application of urea with or without nitrification inhibitors (DCD and DMPP) to a soil grown with sugarcane.

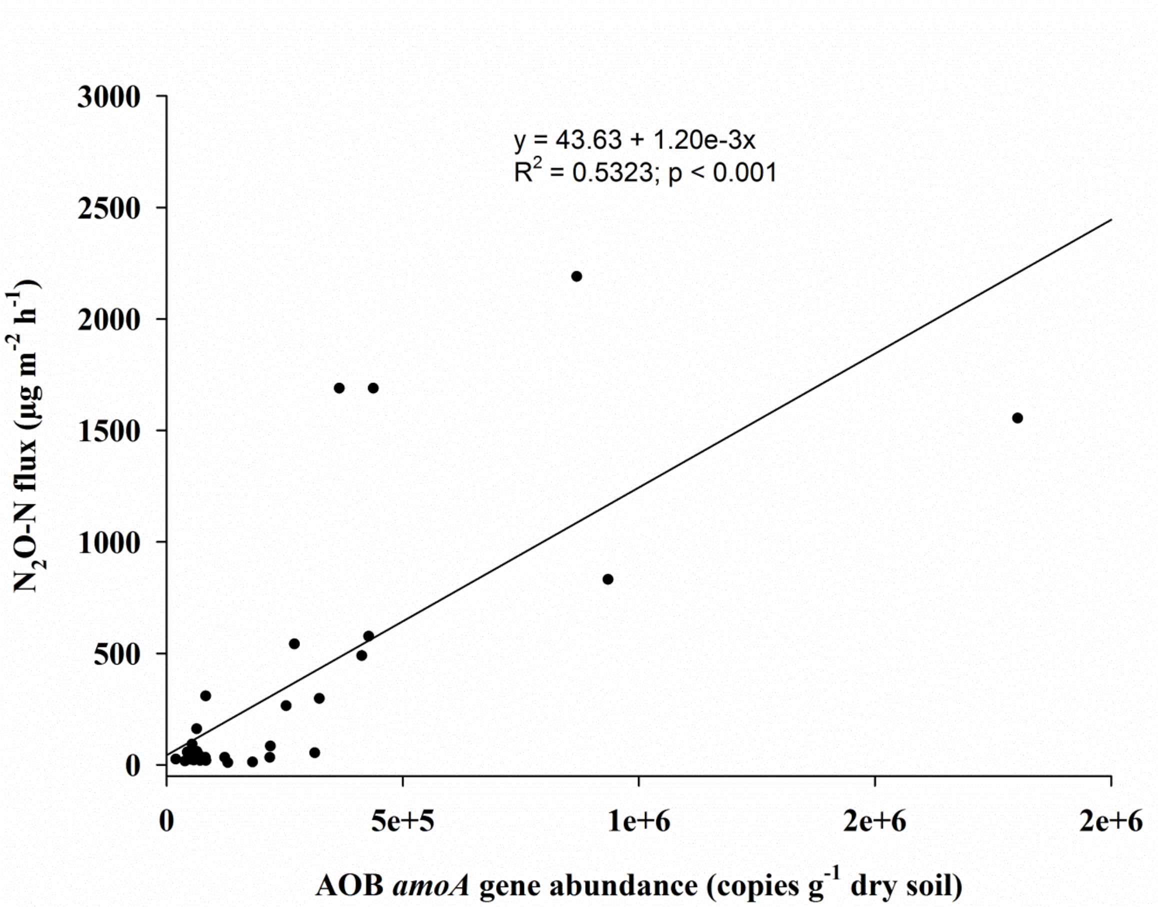

Figure S2. Correlation between ammonia oxidizing bacteria (AOB) *amoA* gene abundance and nitrous oxide emission from a Red Latosol soil 16 days after application of urea with or without nitrification inhibitors (DCD and DMPP), polymer sulfur coated urea (PSCU) and calcium nitrate applied to sugarcane (n = 32).

Figure S3. Rarefaction curves of the microbial communities present in the Red Latosol under treatments with urea, incorporated, with or without nitrification inhibitors (DCD and DMPP); polymer sulfur coated urea (PSCU) and calcium nitrate applied to sugarcane. Time points are separated such that A, B, C, D, E, F, G and H correspond to days 7, 16, 18, 27, 35, 42, 82 and 158 of the experiment.

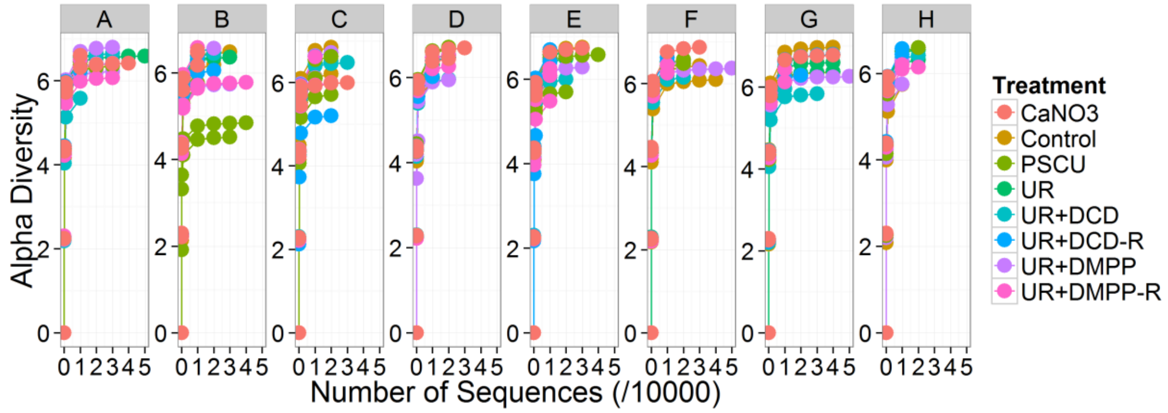

Table S5. Shannon indices and Between-Class Analysis (BCA) ordinations of the bacterial communities under treatments of urea, incorporated in the Red Latosol, with or without nitrification inhibitors (DCD and DMPP); polymer sulfur coated urea (PSCU) or calcium nitrate applied to ratoon sugarcane.

Treatment	16S rRNA gene diversity (Shannon index)		BCA ordination based on 16S gene abundances within Phyla	BCA ordination based on 16S gene abundances within Genus
Treatment	16S rRNA gene diversity (Shannon index)		BCA ordination based on 16S gene abundances within Phyla	BCA ordination based on 16S gene abundances within Genus
7 DAF†				
Control	6.2	ns	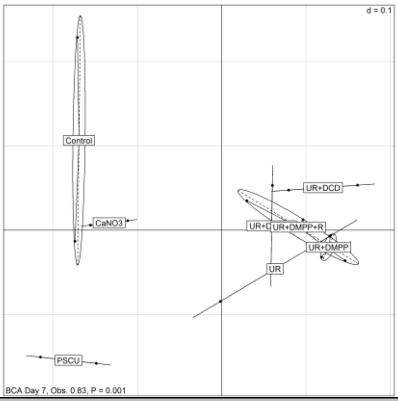	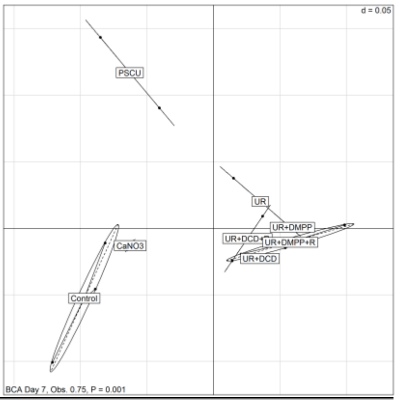
UR	5.8			
UR+DCD - R‡	5.9			
UR+DMPP – R	5.6			
PSCU	5.9			
UR+DCD	5.7			
UR+DMPP	5.7			
Calcium Nitrate	6.3			
16 DAF				
Control	6.1	ns	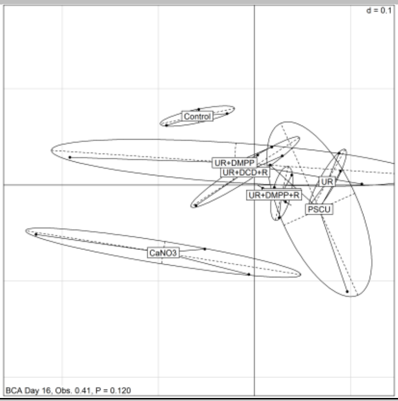	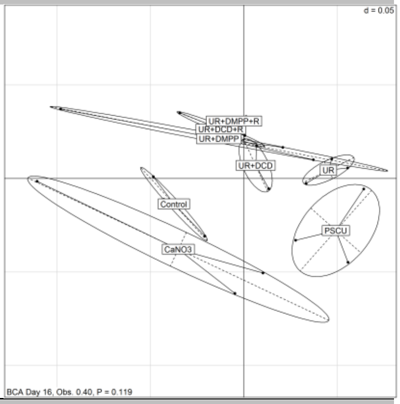
UR	6.0			
UR+DCD - R‡	6.2			
UR+DMPP – R	6.1			
PSCU	5.9			
UR+DCD	6.1			
UR+DMPP	6.0			
Calcium nitrate	6.0			
18 DAF				
Control	6.0	ns	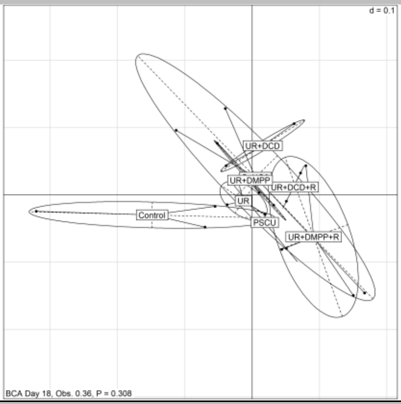	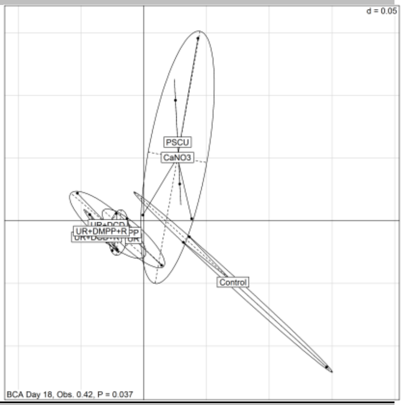
UR	6.0			
UR+DCD - R‡	5.8			
UR+DMPP – R	5.9			
PSCU	5.8			
UR+DCD	5.7			
UR+DMPP	5.9			
Calcium nitrate	5.7			
27 DAF				
Control	6.2	a	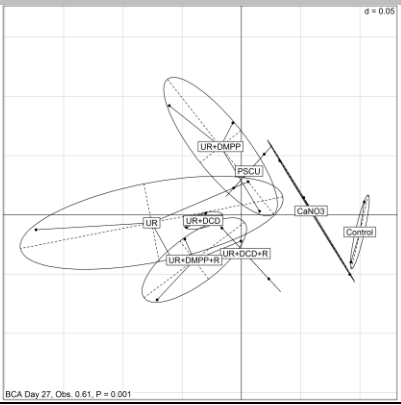	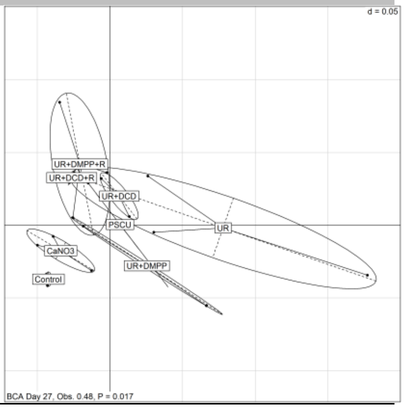
UR	5.3	b		
UR+DCD - R‡	6.0	ab		
UR+DMPP – R	5.8	ab		
PSCU	5.8	ab		
UR+DCD	5.8	ab		
UR+DMPP	5.8	ab		
Calcium nitrate	5.9	ab		

35 DAF				
Control	6.1	*ns*	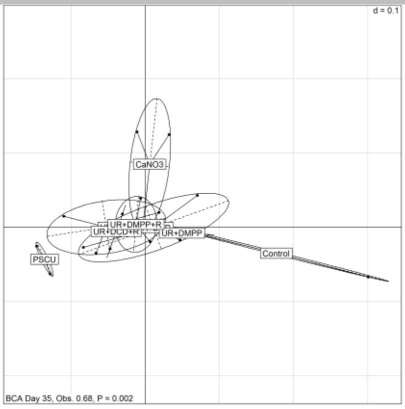	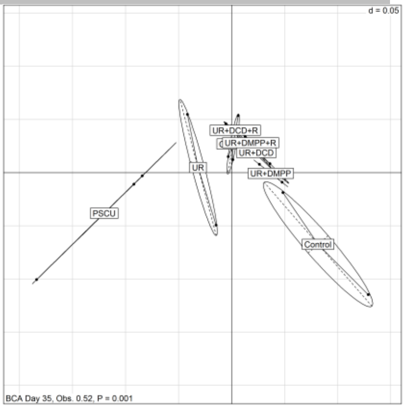
UR	5.7			
UR+DCD - R‡	6.0			
UR+DMPP – R	6.1			
PSCU	5.1			
UR+DCD	5.9			
UR+DMPP	5.5			
Calcium nitrate	5.9			
42 DAF				
No N	6.2	ns	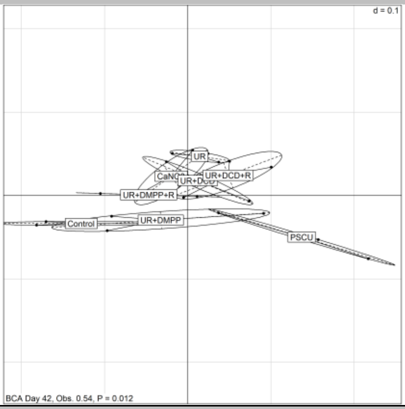	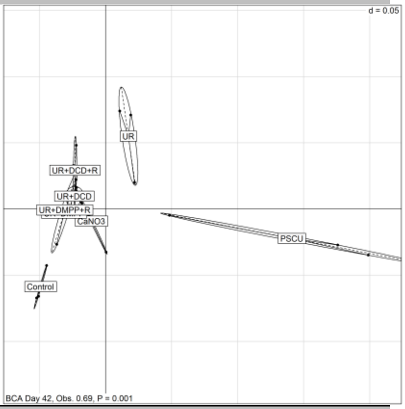
UR	5.8			
UR+DCD - R‡	6.1			
UR+DMPP – R	6.2			
PSCU	5.2			
UR+DCD	6.0			
UR+DMPP	6.1			
Calcium nitrate	6.2			
85 DAF				
Control	6.0	ab	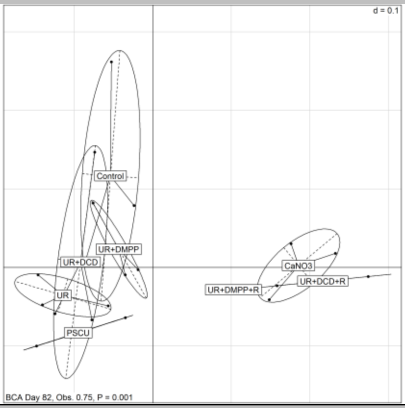	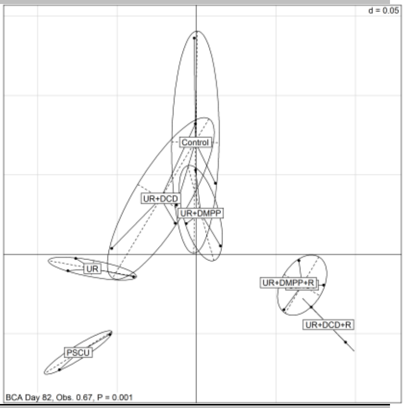
UR	5.6	ab		
UR+DCD - R‡	5.8	ab		
UR+DMPP – R	6.1	ab		
PSCU	5.4	a		
UR+DCD	6.0	ab		
UR+DMPP	6.1	b		
Calcium nitrate	6.0	ab		
158DAF				
Control	6.1	ns	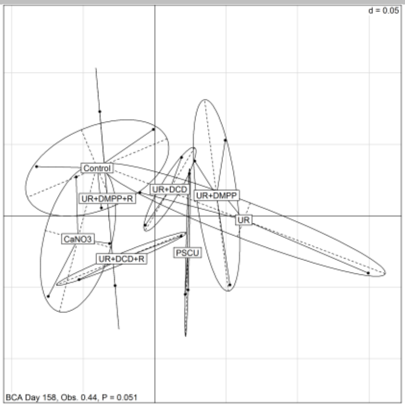	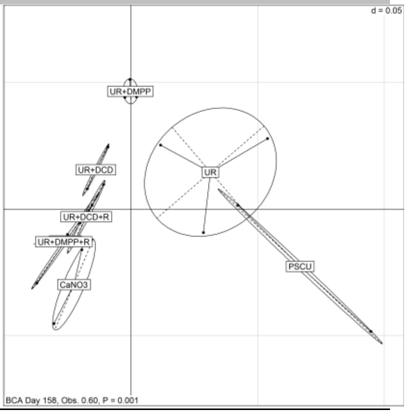
UR	6.0			
UR+DCD - R‡	6.2			
UR+DMPP – R	6.2			
PSCU	5.8			
UR+DCD	6.2			
UR+DMPP	6.1			
Calcium nitrate	6.2			

†Days after fertilizer application

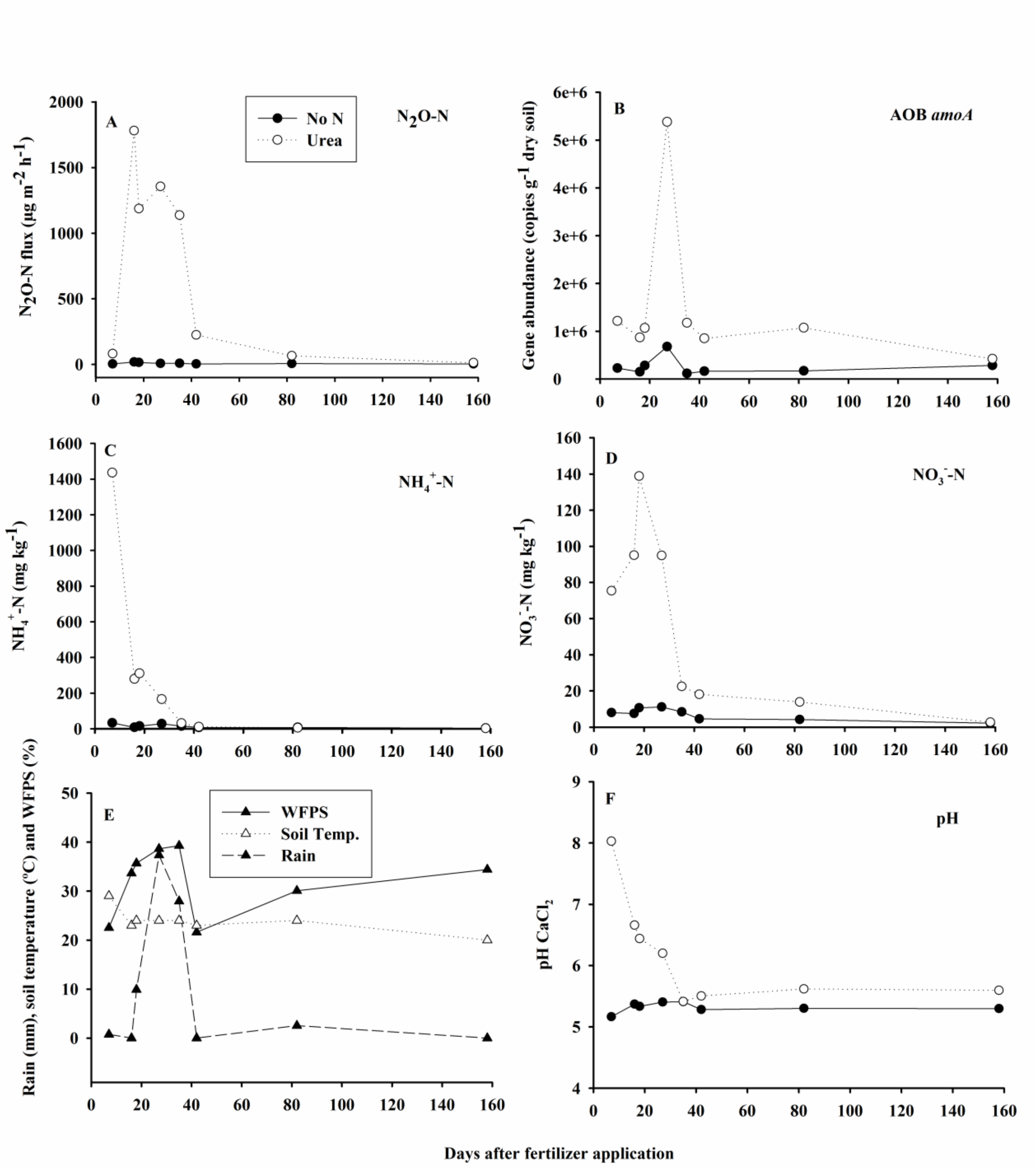

Figure S5. N_2O flux (A), ammonia oxidizing bacteria (AOB) *amoA* gene abundance (B), NH_4^+ (C) and NO_3^- content in soil (D), soil pH (F), rain, soil temperature and water-filled pore space (WFPS) (E) soil pH (F) due urea application to a soil grown with sugarcan as compared to the non-fertilized control.

References

1. Francis, C. A., Roberts, K. J., Beman, J. M., Santoro, A. E. & Oakley, B. B. Ubiquity and diversity of ammonia-oxidizing archaea in water columns and sediments of the ocean. *Proc Natl Acad Sci USA* **102**, 14683–14688 (2005).
2. Rotthauwe, J. H., Witzel, K. P. & Liesack, W. The Ammonia monooxygenase structural gene amoA as a functional marker: molecular fine-Scale analysis of natural ammonia-oxidizing populations. *Appl Environ Microbiol* **63**, 4704 4712 (1997).
3. Henry, S., Bru, D., Stres, B., Hallet, S. & Philippot, L. Quantitative detection of the nosZ gene, encoding nitrous oxide reductase, and comparison of the abundances of 16S rRNA, narG, nirK, and nosZ genes in soils. *App. Environ Microbiol* **72**, 5181–5189 (2006).
4. Henry, S. *et al.* Quantification of denitrifying bacteria in soils by nirK gene targeted real-time PCR. *J Microbiol Methods* **59**, 327–335 (2004).
5. Throbäck, I. N., Enwall, K., Jarvis, A. & Hallin, S. Reassessing PCR primers targeting nirS, nirK and nosZ genes for community surveys of denitrifying bacteria with DGGE. *FEMS Microbiol Ecol* **49**, 401–417 (2004).
6. Fierer, N., Jackson, J. A., Vilgalys, R. & Jackson, R. B. Assessment of soil microbial community structure by use of taxon-specific quantitative PCR assays. *Appl Environ Microbiol* **71**, 4117 – 4120 (2005).
7. Klindworth, A. *et al.* Evaluation of general 16S ribosomal RNA gene PCR primers for classical and next-generation sequencing-based diversity studies. *Nucleic Acids Res* **41**, e1 doi:10.1093/nar/gks808 (2013).
